# P-2192. Hepatitis C Virus Re-infection in the Direct-Acting Antiviral Era within the US Veterans Health Administration

**DOI:** 10.1093/ofid/ofae631.2346

**Published:** 2025-01-29

**Authors:** Bruce Gregoire, Marissa Maier, Lauren Beste, Alexander Matelski, Emily J Cartwright, Elliott Lowy, Timothy R Morgan, Cara D Varley, David Ross

**Affiliations:** Oregon Health & Sciences University, Portland, Oregon; VA Portland Health Care System/Oregon Health and Sciences University, Portland, Oregon; VA Puget Sound Health Care System, Seattle, Washington; Emory University, Atlanta, Georgia; Emory University School of Medicine, Atlanta VA health care system, Decatur, Georgia; VA Puget Sound, Seattle, Washington; VA Long Beach Healthcare System, Long Beach, California; Oregon Health & Science University, Portland, OR; Office of Specialty Care Services, Veterans Health Administration, Washington D.C., District of Columbia

## Abstract

**Background:**

Background and Aims: Hepatitis C virus (HCV) treatment with direct-acting antivirals (DAA) results in a sustained virologic response (SVR) in >90% of individuals (1). Limited prior data describes persons who achieve SVR and later develop recurrent viremia. We identify a cohort with HCV viremia after SVR, detail their risk factors for repeat viremia, and describe the time interval to repeat viremia

Characteristics of Veterans with recurrent HCV viremia (n= 1129)

**Figure:**
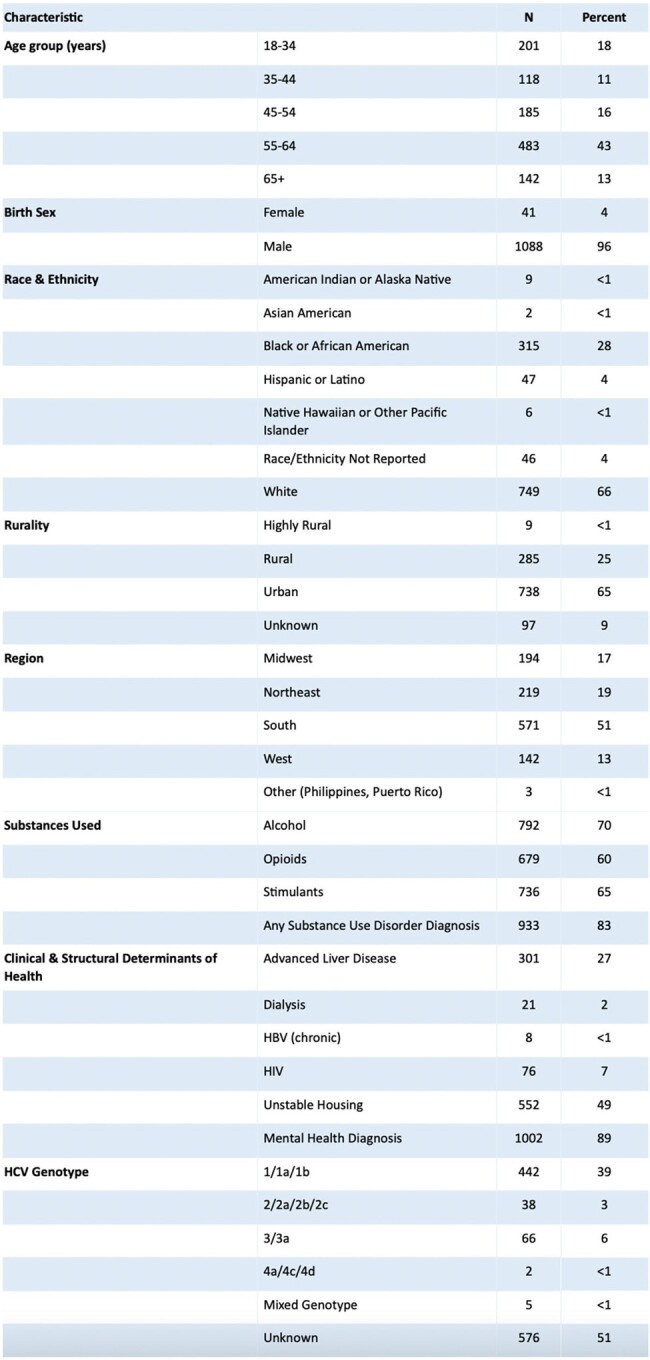

**Methods:**

We identified individuals in the Veterans Health Administration in care during 2021-2022 who received DAAs between January 1, 2014 and December 31, 2022, achieved SVR, and developed repeat viremia. We performed a manual chart review of a randomly selected subset of patients (10%) who were adjudicated into four categories defined as: (1) definite reinfection (HCV genotype change after SVR), (2) presumed reinfection (injection drug use, HIV pre-exposure prophylaxis receipt, new HIV or hepatitis B diagnoses, or incarceration between SVR and repeat viremia), (3) false positive result based on clinician assessment, or (4) neither (possible late relapse).

Adjudication as confirmed reinfection, probable reinfection, false positive, or neither based on chart review, N = 110

**Figure:**
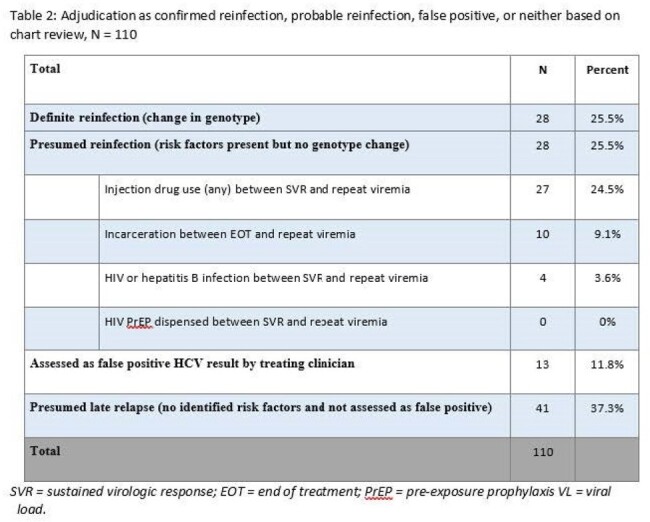

SVR = sustained virologic response; EOT = end of treatment; PrEP = pre-exposure prophylaxis VL = viral load.

**Results:**

Among the 1,129 individuals in the cohort, median time to viremia after SVR was 23 months (IQR 9 – 42 months). Of the 110 individuals randomly selected for chart review, 25.5% (28/110) were definite reinfections, 25.5% (28/110) presumed reinfections, 11.8% (13/110) false positives, and 37.3% (41/110) did not meet any of the above criteria and were considered possible late relapses. Overall, 56/110 (50.9%) had a genotype change or known risk factor for reinfection, with IDU being the most common risk factor (27/110), followed by incarceration (10/110).

Time to repeat viremia

**Figure:**
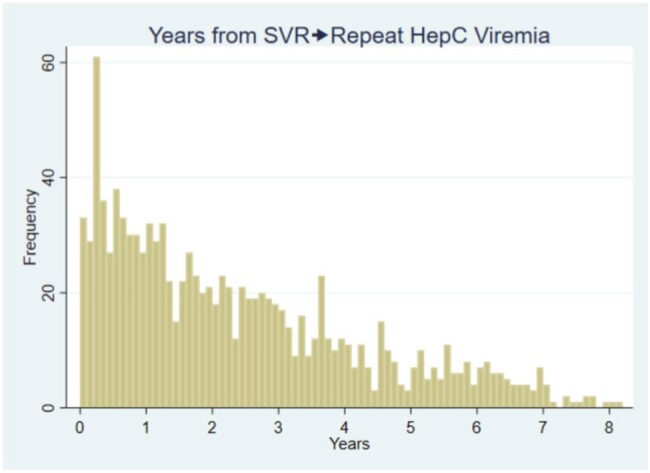

**Conclusion:**

Our study found the median time to repeat viremia is 1.9 years with 75% of individuals developing repeat viremia within 3.6 years. In detailed chart review of a random subset of individuals with repeat viremia, half had confirmed or probable reinfection, and a clinically significant minority were false positive. After EMR review, over one third had no identified risk factor for repeat viremia.

(1) Nguyen V, et al. *JAMA Netw Open.* 2022;5(12).

**Disclosures:**

All Authors: No reported disclosures

